# *Adamdec1*, *Ednrb and Ptgs1*/*Cox1*, inflammation genes upregulated in the intestinal mucosa of obese rats, are downregulated by three probiotic strains

**DOI:** 10.1038/s41598-017-02203-3

**Published:** 2017-05-16

**Authors:** Julio Plaza-Díaz, Cándido Robles-Sánchez, Francisco Abadía-Molina, Virginia Morón-Calvente, María José Sáez-Lara, Alfonso Ruiz-Bravo, María Jiménez-Valera, Ángel Gil, Carolina Gómez-Llorente, Luis Fontana

**Affiliations:** 1 Department of Biochemistry and Molecular Biology II, School of Pharmacy, University of Granada, Granada, Spain; 2 Institute of Nutrition and Food Technology “José Mataix”, Biomedical Research Center, Parque Tecnológico Ciencias de la Salud, University of Granada, Granada, Spain; 3 Instituto de Investigación Biosanitaria ibs.GRANADA, Granada, Spain; 4 Department of Cell Biology, School of Sciences, University of Granada, Granada, Spain; 5 Department of Biochemistry and Molecular Biology I, School of Sciences, University of Granada, Granada, Spain; 6 Department of Microbiology, School of Pharmacy, University of Granada, Granada, Spain; 7 CIBEROBN, Instituto de Salud Carlos III, Madrid, Spain

## Abstract

We have previously reported that administration of *Lactobacillus paracasei* CNCM I-4034, *Bifidobacterium breve* CNCM I-4035 and *Lactobacillus rhamnosus* CNCM I-4036 to obese Zucker-Lepr^*fa*/*fa*^ rats attenuates liver steatosis and exerts anti-inflammatory effects. The goal of the present work was to investigate the modulation of gene expression in intestinal mucosa samples of obese Zucker-Lepr^*fa*/*fa*^ rats fed the probiotic strains using a DNA microarray and postgenomic techniques. We also measured secretory IgA content in the gut and lipopolysaccharide (LPS)-binding protein (LBP) in serum. Expression of three genes (*Adamdec1*, *Ednrb* and *Ptgs1*/*Cox1*) was up-regulated in the intestinal mucosa of the obese rats compared with that in the rats when they were still lean. Probiotic administration down-regulated expression of *Adamdec1* and *Ednrb* at the mRNA and protein levels and that of *Ptgs1*/*Cox1* at the mRNA level, and this effect was in part mediated by a decrease in both macrophage and dendritic cell populations. Probiotic treatment also increased secretory IgA content and diminished the LBP concentration. Based on results reported in this work and else where, we propose a possible mechanism of action for these bacterial strains.

## Introduction

Metabolic syndrome, better referred to as insulin resistance syndrome (IRS), was originally defined as concomitant hyperlipidemia, hypertension, insulin resistance and obesity^1, 2^. IRS often precedes the onset of type 2 diabetes and increases the risk of cardiovascular disease^3, 4^; accordingly, IRS has reached pandemic levels and become a major public health concern. The Zucker rat shows many of the features of IRS; therefore, it is one of the most commonly used genetic models of this syndrome^4^. Zucker-Lepr^*fa*/*fa*^ rats exhibit obesity, hyperglycemia, insulin resistance, hypercholesterolemia, hypertriglyceridemia, and elevated serum free fatty acid concentrations in contrast to Zucker lean Lepr^+/*fa*^ rats. In addition, Zucker-Lepr^*fa*/*fa*^ rats have hepatic steatosis, as well as elevated serum AST and ALT activities, indicating that the liver component of IRS is also present in this model^5^.

Probiotics are live microorganisms that, when consumed in adequate amounts, confer a health effect on the host^6^. Beneficial effects of probiotics have been reported in allergy, intestinal-related diseases, chronic liver disease, urinary tract infections and respiratory infections, among others^7^. Lactobacilli and bifidobacteria are the genera most frequently used as probiotics. A variety of mechanisms underlying their beneficial effects have been proposed: modification of the gut microbiota, competitive adherence to the mucosa and epithelium, strengthening of the gut epithelial barrier and modulation of the immune system to convey an advantage to the host^8^.

Some authors have described the modulation of gene expression by probiotics. Dykstra *et al*.^9^ reported the induction of the gene coding for mucin 3 (*Muc3*) in the small intestine of rats fed *Lactobacillus plantarum* 299 v, *Lactobacillus rhamnosus* R0011, or *Bifidobacterium bifidum* R0071. Ohtsuka *et al*.^10^ administered *Bifidobacterium breve* M-16V to rat pups during the newborn period and found a lower expression of various inflammation-related genes in the colon.

We have previously reported that the administration of three probiotic strains (*Lactobacillus paracasei* CNCM I-4034, *Bifidobacterium breve* CNCM I-4035 and *Lactobacillus rhamnosus* CNCM I-4036) to healthy human volunteers for 30 days is totally safe^11^ and that their administration for the same period of time to obese Zucker rats attenuates the accumulation of fat in the rats’ liver and exerts anti-inflammatory effects such as lower serum concentrations of tumor necrosis factor (TNF)-α, interleukin (IL)-6 and bacterial lipopolysaccharide (LPS)^5^.

The goal of the present study was to investigate whether these bacterial strains may modulate the gene expression of the intestinal mucosa. For this purpose and with the help of DNA microarray technology, we began by studying the modulation of a great number of genes in intestinal mucosa samples from obese Zucker rats. Subsequent validation of candidate genes by postgenomic techniques narrowed the number of genes affected by the three probiotic strains down to 12. Of these 12 genes, we focused on 3: ADAM-like protein decysin-1 (*Adamdec1*, a gene encoding a metalloprotease whose expression increases in dendritic cell maturation), endothelin receptor type B (*Ednrb*, a gene encoding a G-protein-coupled receptor that nonspecifically binds to endothelin-1, -2 and -3, with a potential role in vasoconstriction/vasodilation and cell proliferation), and cyclooxygenase (COX)-1 (*Ptgs1*/*Cox1*, a gene encoding an enzyme that participates in prostaglandin synthesis). These three genes were found to be overexpressed in the intestinal mucosa of obese Zucker-Lepr^*fa*/*fa*^ rats. Our results also show that *L*. *paracasei* CNCM I-4034, *B*. *breve* CNCM I-4035 and *L*. *rhamnosus* CNCM I-4036 were able to inhibit expression of *Adamdec1* and *Ednrb* at the mRNA and protein levels, as well as expression of *Ptgs1*/*Cox1* at the mRNA level, in the intestinal mucosa of the obese Zucker-Lepr^*fa*/*fa*^ rats.

## Results

In this work, we investigated whether the administration of the probiotic strains *Lactobacillus paracasei* CNCM I-4034, *Bifidobacterium breve* CNCM I-4035 and *Lactobacillus rhamnosus* CNCM I-4036 modulate the expression of genes in the intestinal mucosa of obese Zucker-Lepr^*fa*/*fa*^ rats. For this purpose, over 27,000 rat genes were studied using a DNA array. The analysis of the results revealed effects due to the obese phenotype and others due to probiotic administration.

### Intestinal gene expression in the obese phenotype compared with the lean phenotype

We found changes in gene expression due to the “obese” condition, that is, when Zucker-Lepr^*fa*/*fa*^ rats at baseline (when they were still lean) are compared with Zucker-Lepr^*fa*/*fa*^ rats that were fed placebo for 30 days. Zucker-Lepr^*fa*/*fa*^ rats were obese in comparison with Zucker lean Lepr^+/*fa*^ rats after 30 days of feeding with the placebo (Zucker-Lepr^*fa*/*fa*^, 294.4 ± 5.7 g versus Zucker lean Lepr^+/*fa*^, 241.5 ± 5.6 g. P < 0.001).

**Figure 1:**
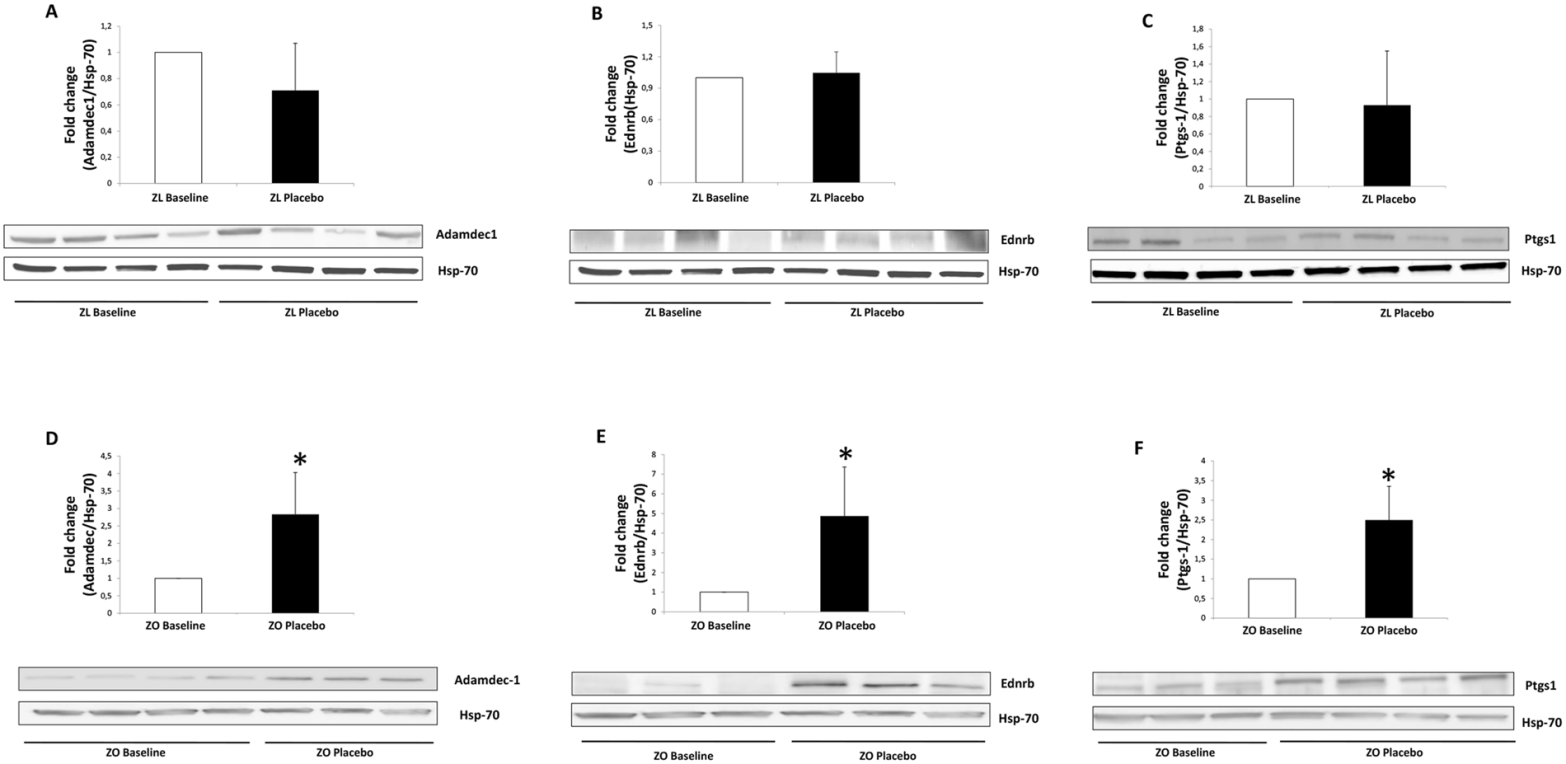
Western blot analysis of Adamdec1, Ednrb and Ptgs1 proteins from Zucker lean Lepr^+/*fa*^ and Zucker-Lepr^*fa*/*fa*^ rats both at baseline and after 30 days of placebo administration. Panels A–C show results for Zucker lean Lepr^+/*fa*^ rats at baseline compared with Zucker lean Lepr^+/*fa*^ rats fed the placebo for 30 days. Panels D–F show results for Zucker-Lepr^*fa*/*fa*^ rats at baseline compared with Zucker-Lepr^*fa*/*fa*^ rats fed the placebo 30 days. The top graph included 7–8 rats per group, and the lower part shows a representative crop blot. Hsp-70 was used as a loading control. ZL, Zucker lean Lepr^+/*fa*^ rats; ZO, Zucker-Lepr^*fa*/*fa*^ rats; *P < 0.05.

**Figure 2:**
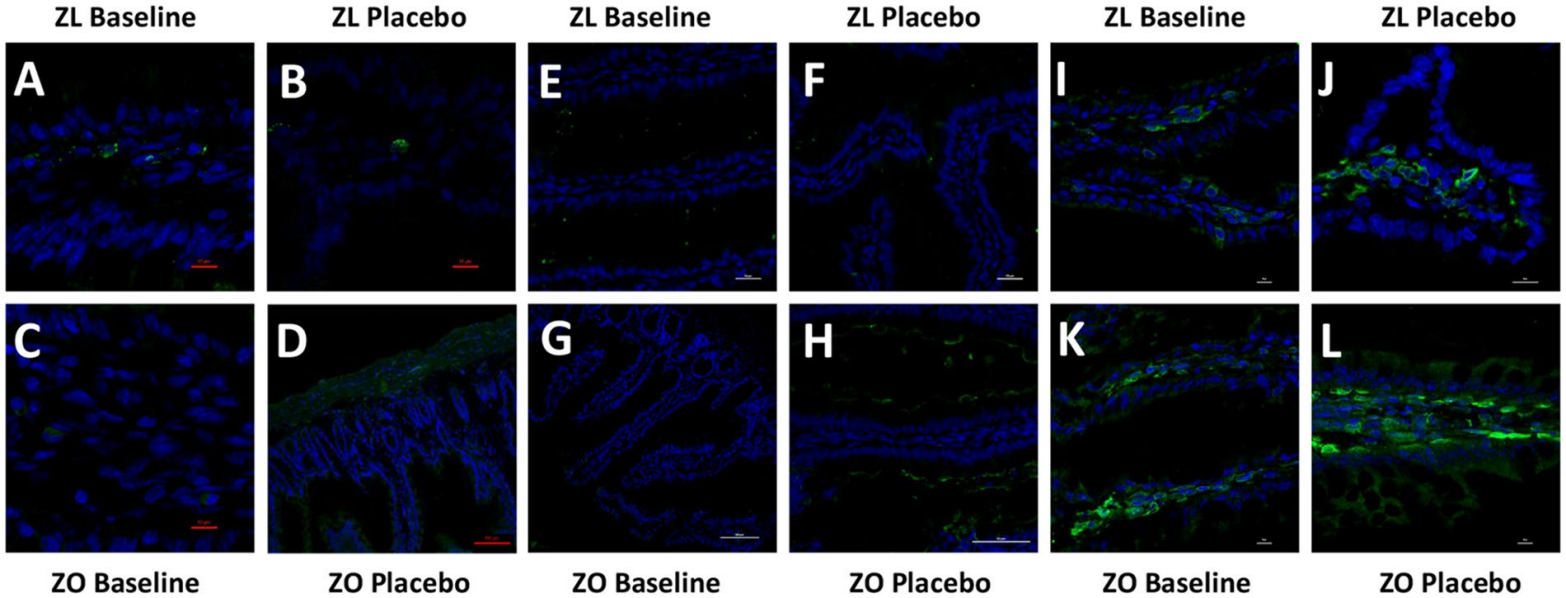
Immunofluorescence analysis of Adamdec1, Ednrb and Ptgs1 proteins from Zucker-lean^+/*fa*^ and Zucker-Lepr^*fa*/*fa*^ rats both at baseline and after 30 days of placebo administration. Panels A–D show results for Adamdec1. Panels E–H show results for Ednrb. Panels I–L show results for Ptgs1. The green color corresponds to Alexa Fluor 488 dye. ZL, Zucker lean Lepr^+/*fa*^ rats; ZO, Zucker-Lepr^*fa*/*fa*^ rats. n = 4 rats per group.

Expression of 6 genes changed when the rats turned obese (Supplementary Table 1). Three of the 6 genes became induced, namely, *Adamdec1*, *Ednrb* and *Ptgs1*/*Cox1*. Protein levels were studied by western blotting and immunofluorescence (Figs 1 and 2). Whereas administration of the placebo did not affect the levels of the proteins encoded by these 3 genes in Zucker lean Lepr^+/*fa*^ (Fig. 1, panels A–C, and Fig. 2), they were increased 3-, 5- and 2.5-fold, respectively, in obese Zucker-Lepr^*fa*/*fa*^ after 30 days of feeding with the placebo (Fig. 1, panels D–F, and Fig. 2).

### Gene expression changes after probiotic administration

The rats that received the mixture of *L*. *paracasei* CNCM I-4034 and *Bifidobacterium breve* CNCM I-4035 exhibited changes in 162 sequences in the intestinal mucosa; the rats that received exclusively *Bifidobacterium breve* CNCM I-4035 had changes in 211 sequences; administration of *L*. *paracasei* CNCM I-4034 modified 663 sequences; and *L*. *rhamnosus* CNCM I-4036 administration changed 404 sequences. All data were compared with those from the Zucker-Lepr^*fa*/*fa*^ rats that received placebo for 30 days (Supplementary Figure 2).

The results of the array showed changes in the expression of 40 genes for *L*. *paracasei* CNCM I-4034; 12 genes for *B*. *breve* CNCM I-4035; 24 genes for *L*. *rhamnosus* CNCM I-4036; and 3 genes for the mixture of *L*. *paracasei* CNCM I-4034 and *B*. *breve* CNCM I-4035. We discarded those genes that i) were not up- or down-regulated ≥ 1.5-fold compared with Zucker-Lepr^*fa*/*fa*^ rats that received a placebo instead of a probiotic strain, and ii) were not modulated by at least two of the probiotic strains (Supplementary Table 2).

qRT-PCR was subsequently used to validate the DNA array data. We focused on three genes that fulfilled the aforementioned criteria, *Adamdec1*, *Ednrb* and *Ptgs1*. All three mRNA levels significantly decreased due to probiotic administration (Supplementary Table 3).

**Figure 3:**
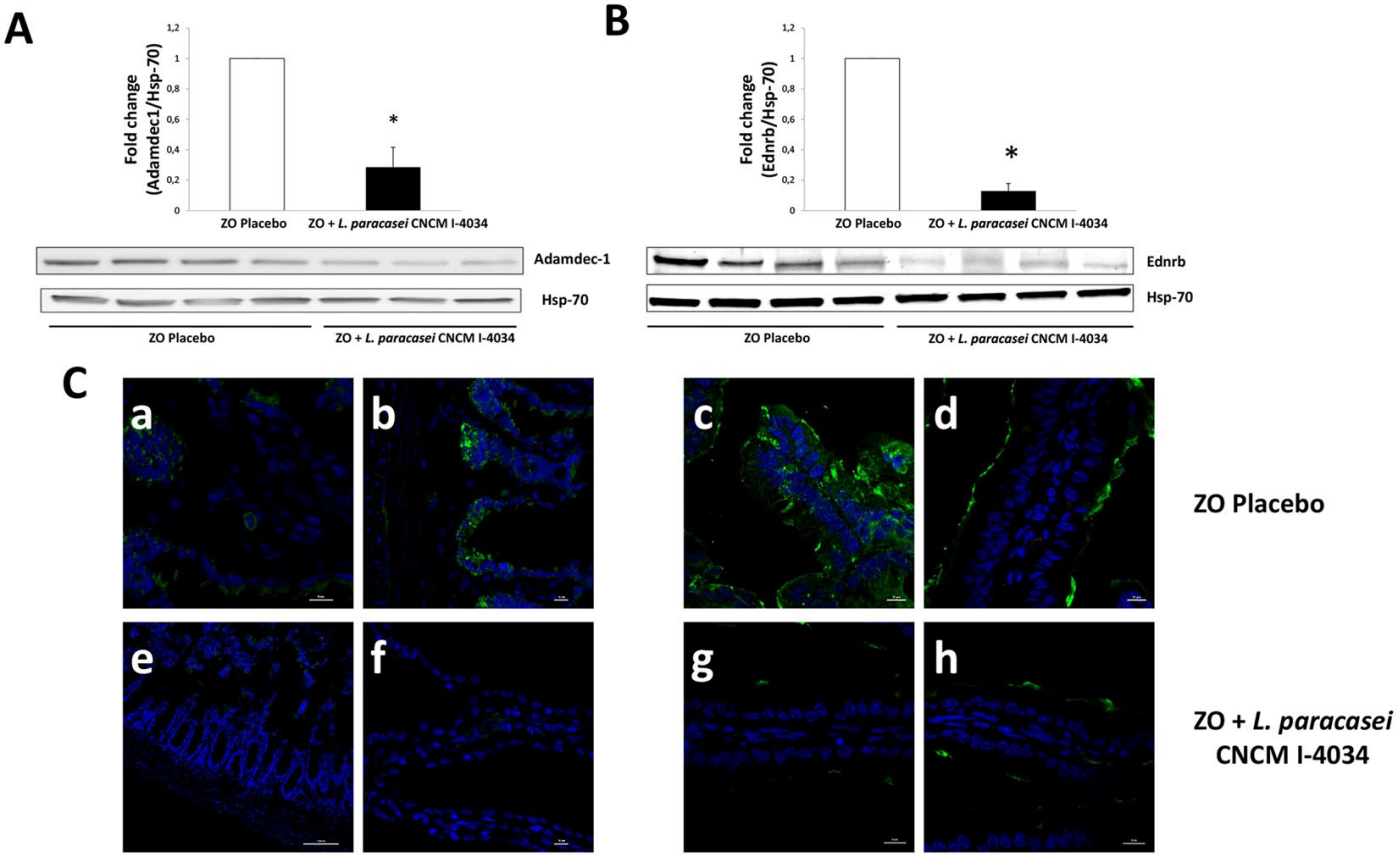
Immunofluorescence and western blot analysis of Adamdec1 and Ednrb proteins from Zucker-Lepr^*fa*/*fa*^ rats fed the placebo compared with Zucker-Lepr^*fa*/*fa*^ rats fed *L*. *paracasei* CNCM I-4034. In panels A and B, top graphs include 7–8 rats per group, and the lower parts show a representative crop blot. Hsp-70 was used as a loading control. *p < 0.05. In panel C, micrographs a,b (ZO placebo), and e,f (ZO + *L*. *paracasei* CNCM I-4034) show results for Adamdec1, and micrographs c,d (ZO placebo), and g,h (ZO + *L*. *paracasei* CNCM I-4034) show results for Ednrb. The green color corresponds to Alexa Fluor 488 dye. ZO, Zucker-Lepr^*fa*/*fa*^ rats.

**Figure 4:**
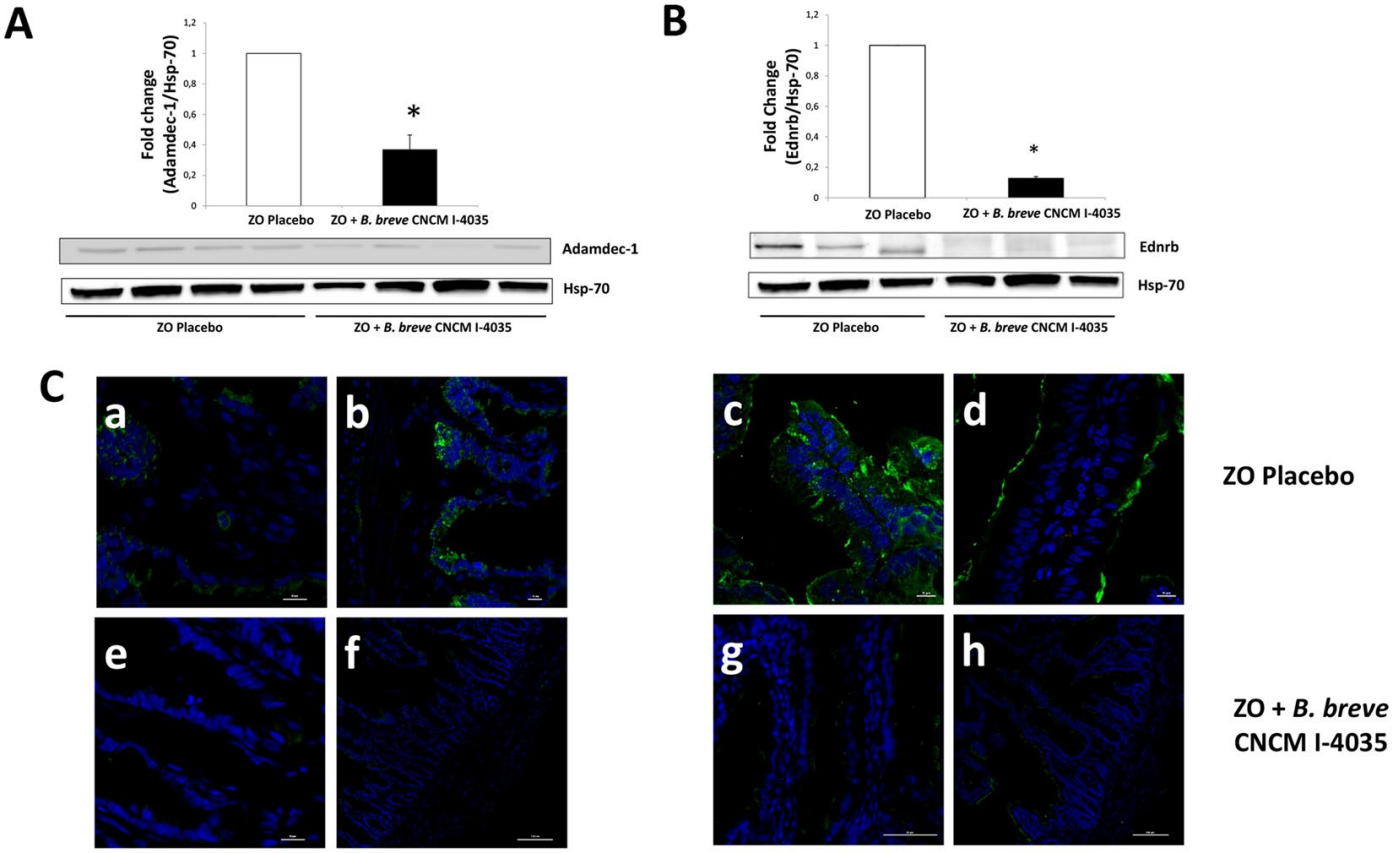
Immunofluorescence and western blot analysis of Adamdec1 and Ednrb proteins from Zucker-Lepr^*fa*/*fa*^ rats fed the placebo compared with Zucker-Lepr^*fa*/*fa*^ rats fed *B*. *breve* CNCM I-4035. In panels A and B, top graphs include 6–8 rats per group, and the lower parts show a representative crop blot. Hsp-70 was used as a loading control. *P < 0.05. In panel C, micrographs a,b (ZO placebo) and e,f (ZO + *B*. *breve* CNCM I-4035) show results for Adamdec1, and micrographs c,d (ZO placebo) and g,h (ZO + *B*. *breve* CNCM I-4036) show results for Ednrb. The green color corresponds to Alexa Fluor 488 dye. ZO, Zucker-Lepr^*fa*/*fa*^ rats.

**Figure 5:**
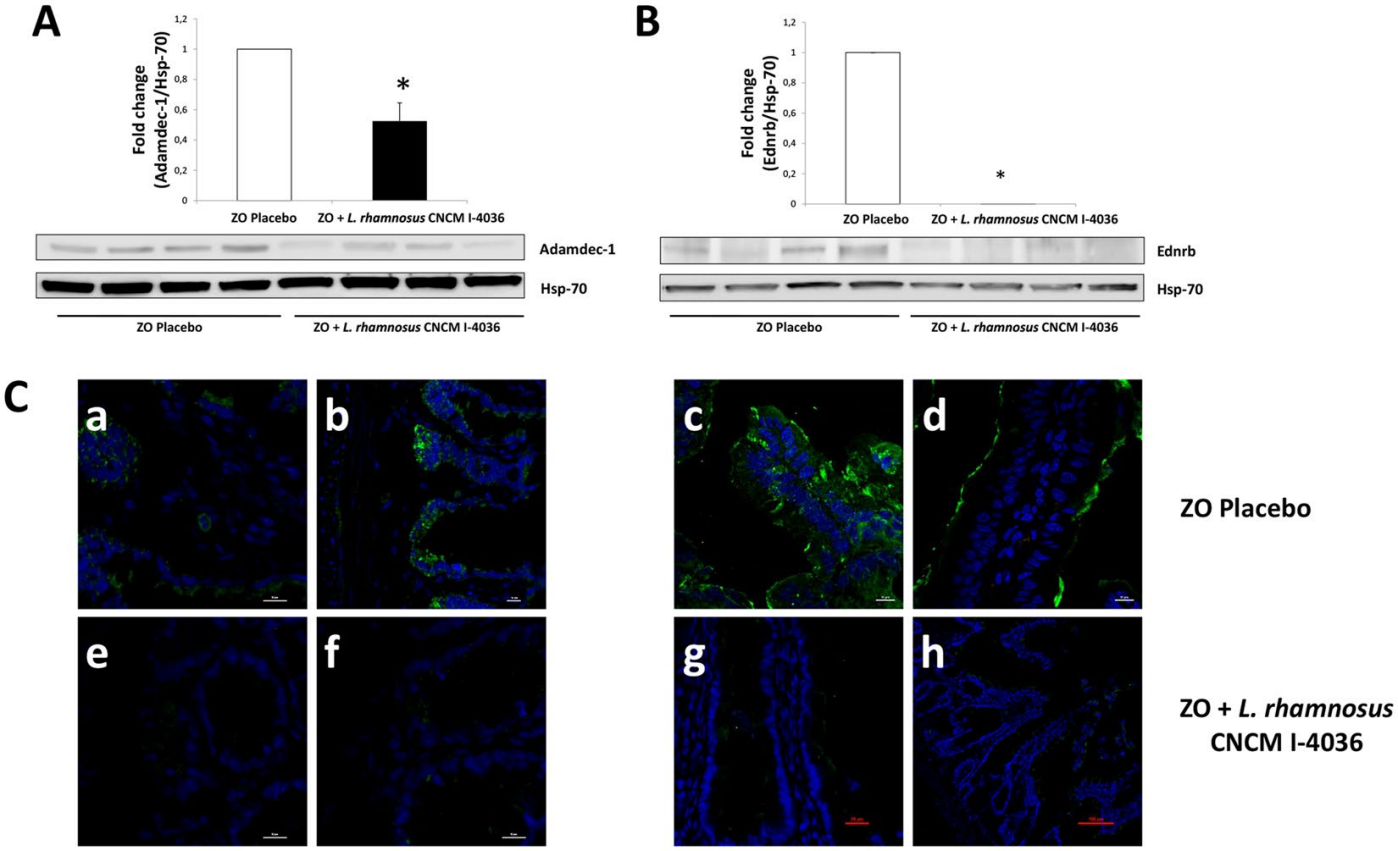
Immunofluorescence and western blot analysis of Adamdec1 and Ednrb proteins from Zucker-Lepr^*fa*/*fa*^ rats fed the placebo compared with Zucker-Lepr^*fa*/*fa*^ rats fed *L*. *rhamnosus* CNCM I-4036. In panels A and B, top graphs include 8 rats per group, and the lower parts show a representative crop blot. Hsp-70 was used as a loading control. *P < 0.05. In panel C, micrographs a,b (ZO placebo) and e,f (ZO + L. *rhamnosus* CNCM I-4036) show results for Adamdec1, and micrographs c,d (ZO placebo) and g,h (ZO + *L*. *rhamnosus* CNCM I-4036) show results for Ednrb. The green color corresponds to Alexa Fluor 488 dye. ZO, Zucker-Lepr^*fa*/*fa*^ rats.

Our next step was to study protein levels corresponding to these genes by both western blot and immunofluorescence techniques. Administration of the probiotic strains for 30 days to obese Zucker-Lepr^*fa*/*fa*^ rats significantly decreased the levels of Adamdec1 and Ednrb proteins in the rats’ intestinal mucosa compared with Zucker-Lepr^*fa*/*fa*^ rats that received the placebo, as shown for *L*. *paracasei* CNCM I-4034 in Fig. 3 (the two proteins decreased 4- and 7-fold, respectively); for *B*. *breve* CNCM I-4035 in Fig. 4 (the two proteins decreased 2.4- and 7-fold, respectively); and for *L*. *rhamnosus* CNCM I-4036 in Fig. 5 (the two proteins decreased 2- and 12-fold, respectively). Ptgs1 protein levels remained unchanged after the administration of the bacterial strains (not shown).

**Figure 6:**
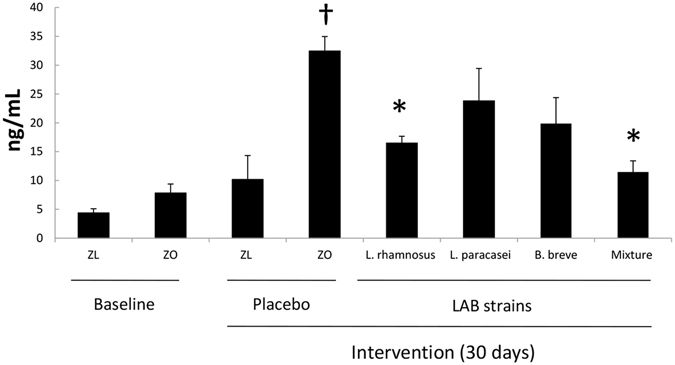
Lipopolysaccharide binding protein (LBP) concentrations in serum of Zucker lean Lepr^+/*fa*^ rats and Zucker-Lepr^*fa*/*fa*^ rats that were fed either a placebo or probiotic strains for 30 days. Values are expressed as the mean ± SEM, n = 8 rats per group. ^†^ *P* < 0.05 versus ZL placebo, and *P < 0.05 versus ZO placebo. ZL, Zucker lean Lepr^+/*fa*^ rats; ZO, Zucker-Lepr^*fa/fa*^ rats.

As an additional effect on the intestine, the content of secretory IgA was measured in the feces of the various groups of rats. Administration of probiotic strains, either alone or in combination, induced an increase in the content of secretory IgA in Zucker-Lepr^*fa*/*fa*^ rats compared with the rats that received the placebo, although this increase was statistically significant only for the *L*. *paracasei* CNCM I-4034 group (2-fold increase, Supplementary Figure 3). Likewise, since probiotic administration has been reported to induce a decrease in bacterial LPS in the rats’ serum^5^, we decided to measure LPS-binding protein (LBP) concentration. These results appear in Fig. 6, which shows that LBP decreased in the serum of the rats fed probiotic strains.

### The obese Zucker-Lepr^*fa*/*fa*^ phenotype is characterized by increases in the number of proinflammatory macrophages and dendritic cells, and the probiotic treatment reversed such effects

To investigate whether the effects of probiotic administration were mediated by changes in the cell population of the intestinal mucosa, qRT-PCR for specific markers of macrophages and dendritic cells was performed (Supplementary Figure 4). Expression of F4/80 was 2-fold higher in Zucker-Lepr^*fa*/*fa*^ rats after 30 days of feeding with the placebo compared with baseline, suggesting an increase in the number of macrophages (Supplementary Figure 4A). The number of dendritic cells was also 2-fold higher in the obese rats as indicated by the levels of the specific dendritic cell marker Cd40 (Supplementary Figure 4B). Probiotic administration significantly diminished levels of F4/80 and Cd40 mRNAs. The decreases ranged 2.5- to 5-fold for F4/80 mRNA, and 4- to 5-fold for Cd40 mRNA. These findings indicate that the obese rats suffered intestinal inflammation, which was alleviated by probiotic administration.

## Discussion

We have described elsewhere the isolation of three probiotic strains from the feces of breastfed newborns. The strains were selected based on their probiotic properties, such as adhesion to intestinal mucus, sensitivity to antibiotics and resistance to biliary salts and low pH. They were identified as *Lactobacillus paracasei* CNCM I-4034, *Bifidobacterium breve* CNCM I-4035 and *Lactobacillus rhamnosus* CNCM I-4036^12^ and demonstrated their safety in mice^12^ and humans^11^.

The main findings of this work were: i) the up-regulation of *Adamdec1*, *Ednrb* and *Ptgs1*/*Cox1* in the intestinal mucosa of obese Zucker rats in comparison with their lean controls; and ii) the repression of two of those genes, *Adamdec1* and *Ednrb*, in the intestinal mucosa of obese Zucker rats fed *L*. *paracasei* CNCM I-4034, *B*. *breve* CNCM I-4035 and *L*. *rhamnosus* CNCM I-4036 for 30 days in comparison with obese Zucker rats fed a placebo for the same period of time. Repression occurred at both the mRNA and protein levels.

ADAMDEC1 is a unique member of the ADAM (A Disintegrin And Metalloproteinase) family and is under-expressed in approximately 10% of Crohn disease patients. This protein is almost exclusively found in macrophages and dendritic cells in the small and large bowel lamina propria. Although its main role as a protease is tissue repair, it has also been hypothesized to play a role in immunity. O’Shea *et al*.^13^ recently exposed *Adamdec1* ^−/−^ mice to dextran sodium sulfate or infected them orally with *Citrobacter rodentium* or *Salmonella typhimurium*, finding that the loss of *Adamdec1* rendered mice more susceptible to the induction of bacterial and chemical-induced colitis, as evidenced by increased neutrophil infiltration, greater IL-6 and IL-1β secretion, more weight loss and increased mortality. In the absence of *Adamdec1*, *g*reater numbers of *Citrobacter rodentium* were found in the spleen, suggestive of a breakdown in mucosal immunity that resulted in bacteremia. Although apparently contradictory to our results, the *Adamdec1* ^*−*/*−*^ mouse, a model in which the gene is completely knocked out, is a situation very different from the Zucker rats. The work of O’Shea *et al*.^13^ proves the role of *Adamdec1* in immunity. However, an inflammatory state in the intestine, such as the one that develops in obesity, is characterized by an increased number of macrophages and dendritic cells. In fact, we found a higher expression of both dendritic cells and macrophages in the intestinal mucosa of the obese rats, which might explain *Adamdec1* induction. Probiotic administration diminished the number of macrophages and dendritic cells and therefore helped attenuate the inflammation.

The endothelin family comprises 3 peptides of 21 amino acids each (ET-1, ET-2, and ET-3), which act through the activation of G-protein-coupled receptors. These receptors seem to have relevant functions in pulmonary and arterial hypertension, atherosclerosis, cerebral vasospasm, and inflammatory signals including edema, fever, pain, and leukocyte recruitment^14^. Endothelin receptor B mutant (Ednrb^*−*/*−*^) mice develop Hirschsprung’s disease, an abnormality of the enteric nervous system characterized by a lack of ganglion cells along a variable length of the distal intestine, resulting in the absence of peristalsis in the aganglionic segment and dilation of the colon proximally^15^. The disease is associated with a severe inflammation of the intestinal mucosa. Yildiz *et al*.^15^ studied mucus barrier properties in Ednrb^*−*/*−*^ mice and found that both passive and active transport (by which particles and microbes translocate across the mucosa, respectively) are diminished in the Ednrb^−/−^ compared to the wild-type proximal colon. As was the case for *Adamdec1*, it is likely that *Ednrb* was induced in the inflamed mucosa of the obese Zucker rats and the probiotic strains exerted an anti-inflammatory effect by decreasing macrophage and dendritic cell numbers. The higher content of secretory IgA found in the obese rats treated with probiotic strains compared with those that received the placebo is in support of such anti-inflammatory effect.

Probiotic administration was also found to down-regulate mRNA levels of a gene involved in prostaglandin synthesis, namely, *Ptgs1*/*Cox1*. COX-1 is a member of the myeloperoxidase family present in some cell types such as macrophages and hematopoietic cells, which regulates the inflammatory response^16^. Two COX isoforms have been identified: whereas COX-2 is the inducible form, COX-1 is constitutive. Therefore, it is not surprising that probiotic administration did not affect Ptgs1/Cox1 protein levels.

Our probiotic strains were also found to exert two protective effects: an increase in the production of secretory IgA in the intestine and a decrease in the concentration of serum LBP. The latter result correlates with the lower levels of LPS that we found in the serum of the obese rats treated with all three bacterial strains^5^. Regarding the higher levels of secretory IgA, similar results have been described in healthy subjects^11^.

**Figure 7:**
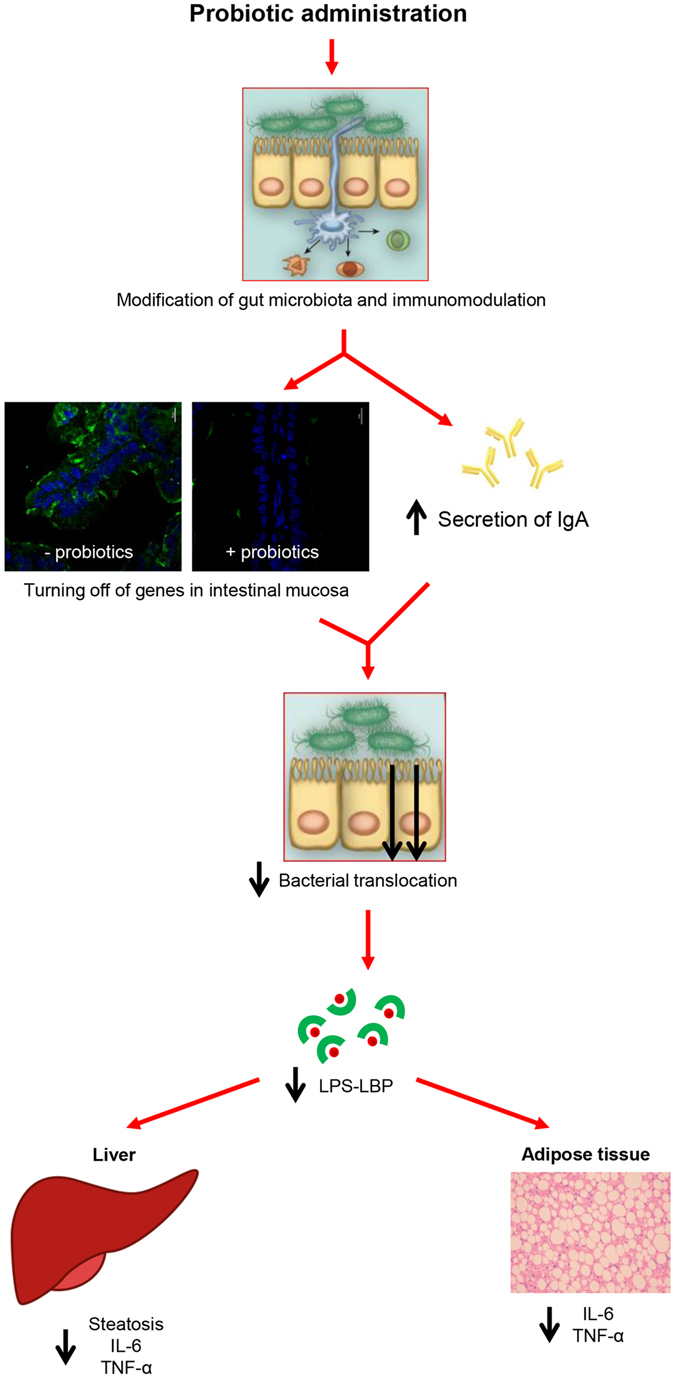
Potential mechanism of action of *L*. *paracasei* CNCM I-4034, *B*. *breve* CNCM I-4035 and *L*. *rhamnosus* CNCM I-4036. LPS: Lipopolysaccharide. LBP: LPS binding protein.

Based on the recapitulation of our results reported in this work and elsewhere, we propose a possible mechanism of action of these bacterial strains (Fig. 7). Administration of *L*. *paracasei* CNCM I-4034, *B*. *breve* CNCM I-4035 and *L*. *rhamnosus* CNCM I-4036 induces changes in gut microbiota composition^11, 17^, which translate into a greater secretion of IgA^11^ and the turning off of *Adamdec1* and *Ednrb* (this work). These changes would result in less bacterial translocation in the intestine and, accordingly, in lower concentrations of LPS-LBP in serum^5^. This, in turn, would have a less severe impact in the liver, which would improve steatosis^5^, and in the white adipose tissue. These two organs would respond by decreasing their production of IL-6 and TNF-α. In fact, we have previously reported low plasma levels of these two cytokines in the Zucker rat model after treatment with the abovementioned probiotics^5^.

Works by other authors support this proposed mechanism of action. A few such works are cited below to support the sequence of events: (1) Reports describing the modification of gut microbiota by probiotics are numerous^18^. For instance, a recent study conducted in high fat diet (HFD)-fed mice by Wang *et al*.^19^ found that probiotic administration shifted the overall structure of the HFD-disrupted gut microbiota toward that of lean mice fed a normal diet. (2) Higher secretion of IgA due to probiotic feeding has been reported in mice by Kemgang *et al*.^20^. (3) In addition to the works by Dykstra *et al*.^9^ and Ohtsuka *et al*.^10^ mentioned before, the study by Tannock *et al*.^21^ can be cited as an example of gene modulation by probiotics. These authors have described the up-regulation of three apoptosis-related genes in the small bowel of mice treated with *L*. *rhamnosus* HN001. (4) The lesser bacterial translocation in the gut might be due to a greater integrity of the mucosal tight junctions. Thus, Balakumar *et al*.^22^ have recently described increases in occludin and zonula occludens-1 transcriptional levels in mice with HFD-induced obesity that received *L*. *rhamnosus* GG, *L*. *plantarum* MTCC5690 and *L*. *fermentum* MTCC5689. Also, supernatants from *Faecalibacterium prausnitzii* and *Escherichia coli* Nissle 1917 have been shown to increase crypt depth in the jejunum of rats with 5-fluorouracil-induced mucositis^23^. (5) Two strains of *Lactobacillus* have been found to lower circulating IgA in HFD-fed rats^24^. (6) Many works describe an attenuation of hepatic steatosis by probiotic administration in mice and rats^25^, as well as a modulation of cytokine production.

In summary, we report that administration of the probiotic strains *L*. *paracasei* CNCM I-4034, *B*. *breve* CNCM I-4035 and *L*. *rhamnosus* CNCM I-4036 to obese Zucker rats increased the content of secretory IgA in the gut, diminished the LBP concentration in the serum, and normalized expression of *Adamdec1* and *Ednrb* at the mRNA and protein levels and that of *Ptgs1*/*Cox1* at the mRNA level in the intestinal mucosa, previously up-regulated in obese Zucker rats.

## Materials and Methods

### Microorganisms

The probiotic strains *Lactobacillus paracasei* CNCM I-4034, *Bifidobacterium breve* CNCM I-4035, and *Lactobacillus rhamnosus* CNCM I-4036 have been characterized and are described elsewhere^12^. These strains were deposited in the Collection Nationale de Cultures de Microorganismes (CNCM) of the Institute Pasteur^12^.

### Ethical statement

This study was conducted in strict accordance with the recommendations in the guidelines for animal research of the University of Granada (Spain). All animals received humane care. The protocol was approved by the Committee on the Ethics of Animal Experiments of the University of Granada (Permit Number CEEA: 2011–377).

### Experimental design

Forty-eight Zucker-Lepr^*fa*/*fa*^ and 16 Zucker lean Lepr^+/*fa*^ male rats weighing 168–180 g were purchased from Harlan Laboratories (Charles River, Barcelona, Spain). The rats were housed in metabolic cages with a 12-h light-dark cycle and had free access to water and food. After 5 days of adaptation, 8 Zucker lean Lepr^+/*fa*^ and 8 Zucker-Lepr^*fa*/*fa*^ rats were euthanized as a reference (baseline). The remaining 40 Zucker-Lepr^*fa*/*fa*^ rats were then randomly assigned to receive 10^10^ colony-forming units (CFU) of one of the three probiotic strains, a mixture of *Lactobacillus paracasei* CNCM I-4034 and *Bifidobacterium breve* CNCM I-4035, or a placebo by oral administration each day for 30 days. An additional group of 8 Zucker lean Lepr^+/*fa*^ rats received the placebo for 30 days. The placebo contained 67% cow’s milk powder, 32.5% sucrose, and 0.56% vitamin C.

After the intervention, the animals were anesthetized and sedated with ketamine and xylazine. Blood was drawn from the aorta and centrifuged for 10 min at 1000 × *g* and 4 °C to separate the serum from the cells. Samples of intestinal mucosa were also taken.

### DNA microarray

We used Affymetrix Rat Gene 1.1 ST Array Plates (Affymetrix Inc., Santa Clara, CA) following the manufacturer’s directions. Briefly, RNA extraction was performed using the RNeasy Mini Kit and Qiacube system. A quantity of 300 mg total RNA was utilized in cDNA synthesis with the Ambion WT Expression kit, and the resulting cDNA was fragmented using uracil-DNA glycosylase and APE1 (apurinic/apyrimidinic endonuclease-1). The labeling process was performed using the WT Terminal Labeling Kit (Affymetrix) with deoxynucleotidyl transferase linked to biotin. After fragmentation, 5.5 µg cDNA was hybridized using the GeneChip Hybridization, Wash and Stain Kit from Affymetrix. GeneChips were scanned using the GeneTitan™. The data were analyzed with the Command Console (AGCC 3.1, Affymetrix) and the Expression Console (EE 1.1, Affymetrix). The value definition was performed using the RMA (Robust Multichip Average) signal intensity.

The complete data set in the present study complies with the MIAME (Minimum Information About a Microarray Experiment) requirements and was uploaded into the *Gene Expression Omnibus* (GEO) database with the title *Expression data from intestinal mucosa of Zucker rats* and reference GSE73848 (https://www.ncbi.nlm.nih.gov/geo/).

The intensity value of each probe in the array analysis was normalized with the Robust Multichip Average (RMA) using the Partek Genomics Suite version 6.10 (Partek) to obtain an individual intensity for each set of probes. All expression data were averaged to achieve a unique expression value for the gene, and the background was deleted. The identification of expression changes was performed using multiple regression models comparing the intensity of each gene with the interaction (Zucker lean Lepr^+/*fa*^ or Zucker-Lepr^*fa*/*fa*^ rats that received placebo versus Zucker-Lepr^*fa*/*fa*^ rats that received any probiotic strain). The two-dimensional hierarchic cluster with the statistically significant sequences (n = 936) appears in Supplementary Figure 1, according to the intensity and the aforementioned interactions.

### qRT-PCR

For validation of the DNA microarray results we used the RT^2^-Profiler PCR array (SABiosciences Corporation, Frederick, MD), which is a two-step qRT-PCR platform. Briefly, total RNA was extracted using the RNeasy Mini Kit (Qiagen, Barcelona, Spain) according to the manufacturer’s recommendations. Isolated RNA was then treated with the RNase-Free DNase Set (Qiagen, Barcelona, Spain). The final RNA concentration and quality were determined using a NanoDrop 2000 spectrophotometer (NanoDrop Technologies, Winooski, Vermont, USA). The cDNA was synthesized from total RNA with an RT^2^ First-Strand Kit (SABiosciences). Real-time qRT-PCR analysis of the samples was performed using a PCR array (SABiosciences), including primer pairs specific for 14 genes involved: *Rgs16*, *Per1*, *Slc7a11*, *Fkbp5*, *Alox5ap*, *Ednrb*, *Adamdec1*, *Ptgs1*, *Tlr9*, *Naip*, *Nfkbia*, and *Nfkb1*. The housekeeping genes *Gapdh* and *Actb* were used as a control. The cDNA was then subjected to real-time PCR with an RT^2^ real-time PCR SYBR green/ROX kit (SABiosciences) on an ABI Prism 7900HT sequence detector (Applied Biosystems, Foster City, CA).

Primers for F4/80 (UniqueAssayID: qRnoCID0007957), Cd40 (UniqueAssayID: qRnoCID0003897), and Gapdh (UniqueAssayID: qRnoCID0057018) were obtained from Bio-Rad Laboratories (Hercules, CA). The qPCR was performed with an ABI Prism 7900 instrument (Applied Biosystems, Foster City, CA, USA) using SYBR Green PCR Master Mix (Applied Biosystems, Foster City, CA, USA).

The PCR conditions were 1 cycle of 95 °C for 10 min followed by 40 cycles of 95 °C for 15 s and 60 °C for 1 min. The expression level of each gene was analyzed with RT^2^ Profiler PCR Array Data Analysis software (version 3.4, SABiosciences). Changes in gene expression were expressed as fold changes (Fc).

### Western blotting

Intestinal mucosa samples were harvested in 10 mM Tris-HCl (pH 7.5), 150 mM NaCl, 2 mM EDTA, 1% Triton X-100, 10% glycerol and a protease inhibitor cocktail (Thermo Scientific) and were placed on ice for 20 min. After centrifugation (30 min, 13,000 × *g*, 4 °C), the protein content in the supernatant was determined using the Protein Assay Kit II (Bio-Rad Laboratories). Samples containing 50 μg protein each were mixed with 3X SDS-PAGE sample buffer (100 mM Tris-HCl, pH 6.8, 25% SDS, 0.4% bromophenol blue, 10% β-mercaptoethanol and 2% glycerol), separated via SDS-PAGE using a TGX Any kD gel (Bio-Rad Laboratories, California, USA) and transferred to a nitrocellulose membrane (Bio-Rad Laboratories, California, USA). After incubation in blocking buffer (5% non-fat milk and 1% Tween 20 in Tris-buffered saline, TBS), the membranes were probed with the following antibodies: anti-Ednrb (1:2000 in 5% bovine serum albumin (BSA), ab117529), anti-Adamdec1 (1:1000 in 5% non-fat milk, NBP1-59146) anti-Ptgs1 (1:2000 in 5% BSA, ab109025), and anti-Hsp-70 (internal control; 1:500 in 5% non-fat milk, sc-7298). Immunoreactive signals were detected via enhanced chemiluminescence (SuperSignal West Dura Chemiluminescent Substrate, 34075, Thermo Scientific, Europe), and the membranes were digitally imaged and analyzed using ImageJ software for densitometric analysis. The results are expressed as the fold change in expression relative to the control.

### Immunofluorescence analysis

Intestinal mucosa samples were fixed in Tissue-Tek O.C.T. Compound (Sakura Finetek, USA). Sections 8 µm in thickness were obtained for confocal microscopy analysis. Samples were washed with phosphate-buffered saline (PBS) for 15–20 min at 4 °C. Then, Triton X-100 0.2% in PBS and normal serum goat (NGS) 1:10 were added. The primary antibodies were anti-Ednrb (1:2000 in PBS, ab117529), anti-Adamdec1 (1:250 in PBS, TA323936 Origene), and anti-Ptgs-1 (1:100 in PBS, ab109025). Samples were incubated for 2 h 45 min at room temperature. Sections were washed 3 times with PBS for 5 min per wash. Then, the secondary antibody (anti-rabbit green Alexa 1:1000 in PBS, A11008) was added for 50 min and washed out with PBS plus Hoechst H6024 (Sigma-Aldrich, St. Louis, MO), 1:1000 in PBS. Finally, Dako (Agilent Product Solutions, Barcelona, Spain) was used to fix sections with cover slips (Menzel-Glaser, 24 × 60 mm #1, Denmark).

### Serum lipopolysaccharide binding protein (LBP) concentration

Serum LBP was measured with an enzyme-linked immunosorbent assay kit from Cloud-Clone Corp. (Houston, USA), following the manufacturer’s directions.

### Determination of the fecal content of secretory IgA

Secretory IgA was analyzed in feces by enzyme-linked immunosorbent assay (Immundiagnostik AG, Bensheim, Germany) according to the manufacturer’s instructions.

### Statistical analysis

All results are expressed as the mean ± SEM. Differences between effects at baseline and after 30 days for Zucker lean Lepr^+/*fa*^ or Zucker-Lepr^*fa*/*fa*^ rats were assessed by either parametric (unpaired Student’s t-test) or non-parametric (Mann-Whitney *U*-test) tests. Differences for any variable between Zucker-Lepr^*fa*/*fa*^ rats that received placebo and any group of Zucker-Lepr^*fa*/*fa*^ rats that received a specific strain were analyzed using a one-way ANOVA and *a posteriori* Bonferroni test. *P* < 0.05 was considered statistically significant. All analyses were performed using the statistical package IBM SPSS (Statistical Package for the Social Sciences) Statistics version 20 (Somers, NY).

## Electronic supplementary material

Supplementary PDF File
