# Supplementary material for: Adamdec1, Ednrb and Ptgs1/Cox1, inflammation genes upregulated in the intestinal mucosa of obese rats, are downregulated by three probiotic strains
Source: Sci Rep. 2017 May 16;7:1939. doi: 10.1038/s41598-017-02203-3 (PMC5434015; doi:10.1038/s41598-017-02203-3)
Supplement: Supplementary file 1 — Supplementary PDF File [file 41598_2017_2203_MOESM1_ESM.pdf]

Title: *Adamdec1, Ednrb and Ptgs1/Cox1*, inflammation genes upregulated in the intestinal mucosa of obese rats, are downregulated by three probiotic strains

Authors: Julio Plaza-Díaz[1,2,3], Candido Robles-Sánchez[1,2,3], Francisco Abadía-Molina[4], Virginia Morón-Calvente[4], Maria Jose Sáez-Lara[2,5], Alfonso Ruiz-Bravo[6], Maria Jiménez-Valera[6], Angel Gil[1,2,3,7], Carolina Gómez-Llorente[1,2,3,7] & Luis Fontana[1,2,3,#]

Institutions: [1]Department of Biochemistry and Molecular Biology II, School of Pharmacy, University of Granada, Spain. [2]Institute of Nutrition and Food Technology “José Mataix”, Biomedical Research Center, Parque Tecnológico Ciencias de la Salud, University of Granada, Spain. [3]Instituto de Investigación Biosanitaria ibs.GRANADA, Spain. [4]Department of Cell Biology, University of Granada, Granada 18071, Spain, Biomedical Research Center, University of Granada, Granada 18016, Spain. [5]Department of Biochemistry and Molecular Biology I, School of Sciences, University of Granada, Spain. [6]Department of Microbiology, School of Pharmacy, University of Granada, Spain. [7]CIBEROBN, Instituto de Salud Carlos III, Madrid, Spain.

[#]**Corresponding author:** Luis Fontana, Ph. D. Dept. Biochemistry and Molecular Biology II, School of Pharmacy, Campus de Cartuja s/n, 18071 Granada, Spain. Phone: 34958242335. Fax: 34958248960. E-mail: fontana@ugr.es

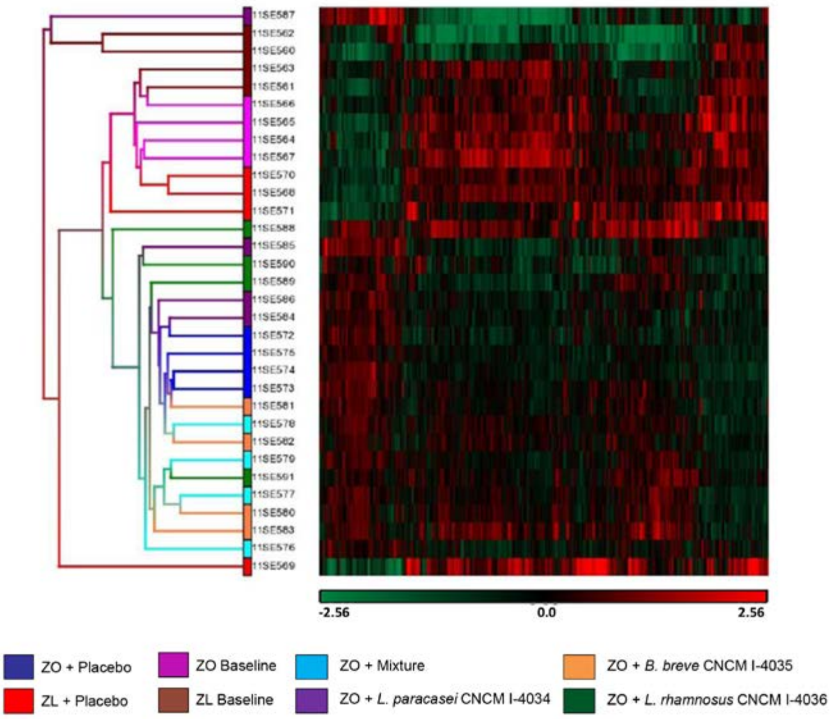

Supplementary Figure 1

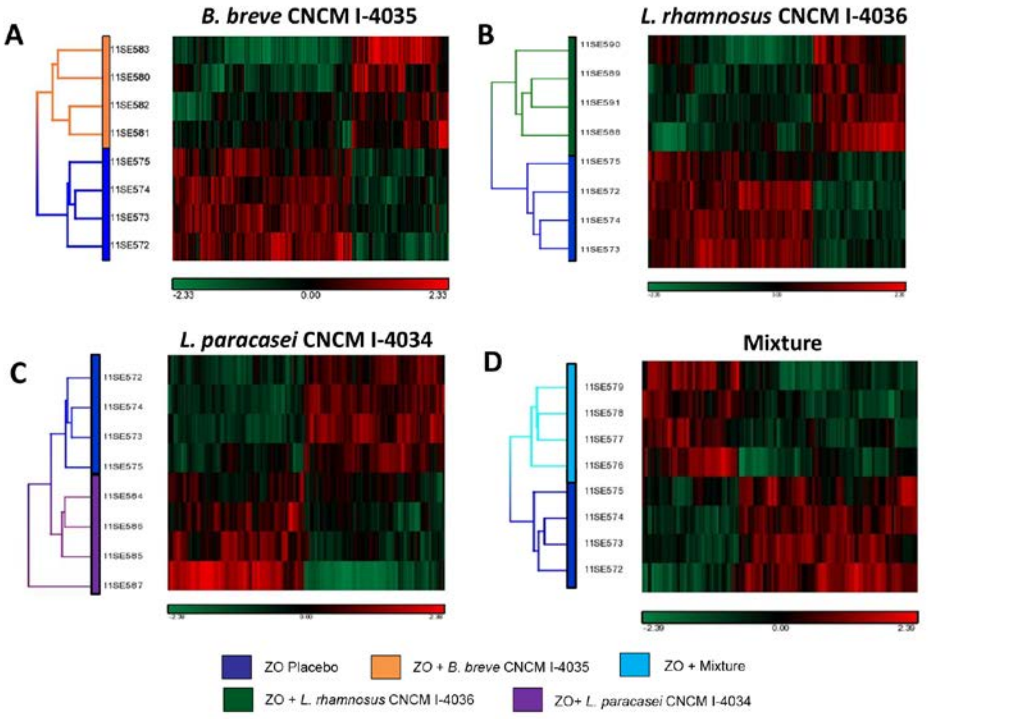

Supplementary Figure 2

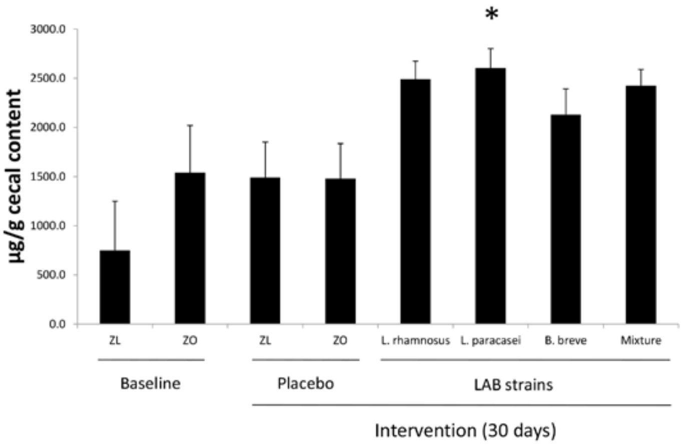

Supplementary Figure 3

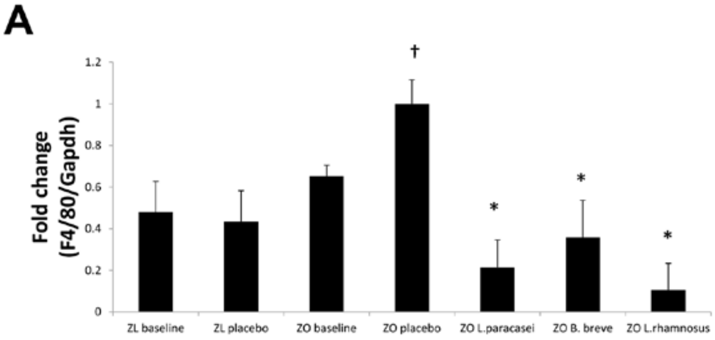

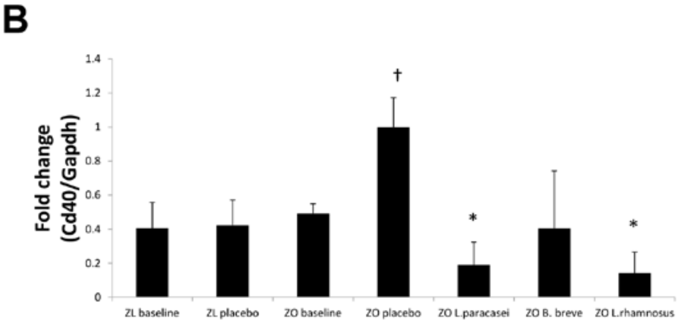

Supplementary Figure 4

Supplementary Figure 1. Microarray results obtained with the Rat Gene 1.1 ST Array Plate (Affymetrix®). These results were uploaded to the Gene Expression Omnibus (GEO) platform as "GSE73848 *Expression data from intestinal mucosa of Zucker rats.*" ZL, Zucker lean Lepr$^{+/fa}$ rats; ZO, Zucker-Lepr$^{fa/fa}$ rats; Mixture, *L. paracasei* CNCM I-4034 plus *B. breve* CNCM I-4035 strains. n=4 rats per group.

Supplementary Figure 2. Microarray expression data from the intestinal mucosa of Zucker-Lepr$^{fa/fa}$ rats treated with *B. breve* CNCM I-4035 **(A)**, *L. rhamnosus* CNCM I-4036 **(B)**, *L. paracasei* CNCM I-4034 **(C)**, or mixture **(D)** compared with Zucker-Lepr$^{fa/fa}$ rats fed the placebo. ZO, Zucker-Lepr$^{fa/fa}$ rats; Mixture, *L. paracasei* CNCM I-4034 plus *B. breve* CNCM I-4035 strains. n=4 rats per group.

Supplementary Figure 3. Total content of secretory IgA in the feces of Zucker-lean$^{+/fa}$ rats and Zucker-Lepr$^{fa/fa}$ rats that were fed either a placebo or probiotic strains for 30 days. Values are expressed as the mean ± SEM, n=8 rats per group. **P*<0.05 versus ZO placebo. ZL, Zucker lean Lepr$^{+/fa}$ rats. ZO, Zucker-Lepr$^{fa/fa}$ rats.

Supplementary Figure 4. Obese rats develop intestinal inflammation, and probiotic treatment mitigates it. qRT-PCR showing mRNA levels for (A) F4/80, and (B) Cd40. Values are expressed as the mean ± SEM, n=8 rats per group. $^{\dagger}P<0.05$ versus ZL baseline, ZL placebo and ZO baseline. **P*<0.05 versus ZO placebo. ZL, Zucker lean Lepr$^{+/fa}$ rats. ZO, Zucker-Lepr$^{fa/fa}$ rats.

Supplementary Table 1. Validation of microarray results for obese Zucker-Lepr$^{fa/fa}$ rats fed the placebo

Intestinal mucosa genes (qRT-PCR)	ZO baseline versus ZO placebo	*P* value
Slc7a11	↓-1.548	0.0315
Fkbp5	↓-1.395	0.0362
Ednrb	↑3.682	0.0001
Adamdec1	↑5.941	<0.001
Ptgs1	↑1.46	0.0101
Nfkbia	↓-1.18	0.0269

This table shows the fold change in gene expression as quantitated by qRT-PCR, as well as the statistical significance (*P* value) compared with Zucker-Lepr$^{fa/fa}$ rats at baseline. Inhibited genes appear as ↓, whereas induced genes appear as ↑. n=8 rats per group.

Supplementary Table 2. List of genes (symbol and reference) whose expression was modulated by the probiotic strains

L. rhamnosus CNCM I-4036 group		*L. paracasei* CNCM I-4034 group			
**gene_assignment**	**Gene Symbol**	**gene_assignment**	**Gene Symbol**	**gene_assignment**	**Gene Symbol**
NM_031345	↓Tsc22d3	NM_017333	↓Ednrb	NM_012521	↑S100g
NM_053551	↓Pdk4	NM_001106046	↓Adamdec1	FQ225348	–
NM_001106611	↓Lsm3	BC091243	↓Igha	BC098746	–
NM_053600	↓Fez2	NM_012802	↓Pdgfra	BC092582	↓LOC314509
ENSRNOT00000065436	↓Grsf1	NM_031048	↓Lifr	ENSRNOT00000040099	↓LOC502822
NM_001108321	↓Rtp4	NM_017272	↓Aldh1a7	*L. paracasei* CNCM I-4034 and *B. breve* CNCM I-4035 groups	
NM_001034012	↓Adamtsl4	ENSRNOT00000057971	↓RGD1564318	**gene_assignment**	**Gene Symbol**
ENSRNOT00000053807	–	BC088423	↓IgG-2a	NM_013096	↑Hba-a2
ENSRNOT00000054185	–	BC097960	↓Igj	NM_013096	↑Hba-a2
ENSRNOT00000052628	–	NM_022407	↓Aldh1a1	NM_001111269	↑LOC689064
ENSRNOT00000053902	–	NM_001007728	↓Mpzl1	NM_198776	↑Hbb-b1
ENSRNOT00000054104	–	L22652	↓IgG-2a	*L. paracasei* CNCM I-4034, *B. breve* CNCM I-4035 and mixture groups	
ENSRNOT00000053447	–	NM_017043	↓Ptgs1	**gene_assignment**	**Gene Symbol**
ENSRNOT00000053929	–	NM_031086	↓Pros1	ENSRNOT00000058247	↓RGD1563231
ENSRNOT00000053402	–	NM_001134469	↓Lrrc49	*L. rhamnosus* CNCM I-4036, *B. breve* CNCM I-4035 and mixture groups	
L. rhamnosus CNCM I-4036 and *L. paracasei* CNCM I-4034 groups		ENSRNOT00000041272	↓RGD1564284	**gene_assignment**	**Gene Symbol**
**gene_assignment**	**Gene Symbol**	NM_001017502	↓Ccdc117	NM_001077589	↓Rgs16
NM_001107673	↓Slc7a11	NM_017260	↓Alox5ap	NM_001034125	↓Per1
NM_001012174	↓Fkbp5	NM_053566	↓Ptch1	*L. rhamnosus* CNCM I-4036 and *B. breve* CNCM I-4035 groups	
ENSRNOT00000053268	–	NM_001009920	↓Gsta5	**gene_assignment**	**Gene Symbol**
ENSRNOT00000053276	–	NM_133583	↓Ndrg2	ENSRNOT00000053105	–

B. breve CNCM I-4035 group		BC088423	↓IgG-2a	ENSRNOT00000052978	–
**gene_assignment**	**Gene Symbol**	NM_001105779	↓Nhp2	FQ233900	–
NM_033234	↑Hbb	NM_022508	↓Mthfd1		
ENSRNOT00000053328	–	NM_031523	↑Klk1b3		

Listed genes were selected from the microarray results based on two criteria: 1) Up- or down-regulation ≥1.5-fold compared with Zucker-Lepr$^{fa/fa}$ rats fed the placebo, and 2) expression regulated by at least two of the probiotic strains. Comparisons were made versus Zucker-Lepr$^{fa/fa}$ rats fed the placebo. Inhibited genes appear as ↓, whereas induced genes appear as ↑. Genes lacking name or symbol are of unknown function (dash). n=4 rats per group.

Supplementary Table 3. Validation of microarray results for obese Zucker-Lepr$^{fa/fa}$ rats that received probiotic strains

Intestinal mucosa genes (qRT-PCR)	Mixture	*P* value	*B. breve* CNCM I-4035	*P* value	*L. rhamnosus* CNCM I-4036	*P* value	*L. paracasei* CNCM I-4034	*P* value
Rgs16	-1.4475		-1.1794		1.3041			
Per1	-1.1103		-1.0346		-1.1194			
Slc7a11	↓-1.562	0.045			-1.5256		-1.4578	
Fkbp5					↓-1.8928	0.0061	↓-1.4582	0.0229
Alox5ap	↓-2.0045	<0.001	↓-1.7617	0.0169	-1.5037		↓-1.6926	0.0016
Ednrb	-1.2308		↓-2.2192	0.0063	-2.3345		↓-1.8782	0.0004
Adamdec1	↓-1.6176	0.0429	↓-3.3604	<0.001	↓-3.3349	<0.001	↓-2.7512	<0.001
Ptgs1	-1.2382		↓-1.6275	0.0004	-1.4031		↓-1.3756	0.006
Tlr9	-1.0266		-1.0109		1.8727	0.14	1.4146	0.0611
Naip	1.0523		↓-1.58	0.0409	1.0592		-1.32	
Nfkbia	1.0191		1.04		1.0348		1.0373	
Nfkb1	-1.003		-1.0792		1.0121		-1.12	

This table shows the fold change in gene expression as quantitated by qRT-PCR, as well as the statistical significance (*P* value) compared with Zucker-Lepr$^{fa/fa}$ rats fed the placebo. Inhibited genes appear as ↓. n=8 rats per group.
